# Laboratory Investigation of Storage Stability and Aging Resistance of Slightly SBS-Modified Bitumen Binders

**DOI:** 10.3390/ma16072564

**Published:** 2023-03-23

**Authors:** Hao Yu, Liantong Mo, Yonghan Zhang, Cong Qi, Yulu Wang, Xiang Li

**Affiliations:** 1 State Key Laboratory of Silicate Materials for Architectures, Wuhan University of Technology, Wuhan 430070, China; 303621@whut.edu.cn (H.Y.); 317577@whut.edu.cn (Y.W.); 330994@whut.edu.cn (X.L.); 2 Shandong Haiyun Asphalt Co., Ltd., Binzhou 371600, China; yonghan.zhang@chambroad.com (Y.Z.); cong.qi@chambroad.com (C.Q.)

**Keywords:** slightly SBS-modified bitumen, storage stability, aging resistance, rheological properties, aging index, brittle failure

## Abstract

Slightly SBS-modified bitumen binders have been applied for the asphalt concrete impermeable layer of pumped storage power stations (PSPSs) in China. However, the storage stability and aging resistance of slightly SBS-modified bitumen are big concerns. In this study, three different types of slightly SBS-modified bitumen binders were evaluated by using a commonly used virgin bitumen and a normal SBS polymer-modified bitumen as references. All of the bitumen binders were subjected to short-term and long-term aging that were simulated by using a 5 h and 24 h thin film oven test (TFOT), respectively. A Fourier transform infrared (FTIR) spectroscopy test, storage stability test, dynamic shear rheological test, stress relaxation test, and direct tensile (DT) test were carried out to obtain insight into the storage stability and aging resistance. FTIR analysis indicated that slightly SBS modified bitumen exhibited serious aging of base bitumen together with higher polymer degradation. The aging indexes obtained from the carbonyl index and the polybutadiene (PB) index can well rank the aging resistance. Slightly SBS-modified bitumen binders had excellent storage stability, and even after a long-term period of 7 days of storage, the complex modulus and phase angle remained fairly constant. The rheological master curves were constructed to investigate the effects of short-term and long-term aging. Slightly modified bitumen binders were well identified by the plateau of the phase angle master curves. The aging resistance was well distinguished by the deviation of the complex modulus master curve using unaged bitumen as a baseline. It was found that three types of slightly SBS-modified bitumen binders exhibited inconsistent aging resistance in terms of rheological aging index. The relative change of the initial instantaneous modulus and the modulus relaxation rate was able to explain the relaxation properties. With respect to the direct tensile test, the increase in stiffness modulus and the loss of ultimate tensile strain can be used to evaluate the susceptibility of bitumen aging. An attempt was made to establish the relationship of the aging index between FTIR analysis, rheological properties, and low-temperature performance. It was found that the relationship among these aging indexes was weak. In general, slightly SBS modified bitumen should be well designed to obtain good aging resistance and low-temperature performance. Highly modified bitumen is foreseen to be promising in the case of extremely low temperatures and long-term durability.

## 1. Introduction

Pumped storage hydropower (PSH) is regarded as one of the most common and well-established types of energy storage technologies in the world. It is also the only currently commercialized technology for long-duration storage that can integrate additional renewable resources. Therefore, it is vital to create a clean, flexible, and reliable energy grid. Accelerating the construction of pumped storage power stations (PSPSs) is of great importance to meet the adjustment demand of a new energy power system in the future of China. Currently, more than 100 PSPSs are under construction in China [1,2].

The asphalt concrete facing is commonly used as the anti-seepage structure for pumped storage reservoirs. The design service life of the asphalt concrete anti-seepage facing is usually more than 50 years. Because the asphalt concrete facing is subjected to a severe environment and is subjected to complex external loadings and a long service life, the problem of structural damage, material aging, and deterioration will occur and thus become a big concern [3]. As a result, the asphalt concrete used for the impermeable layer is required to have long-term aging resistance and durability. The selection of high-quality bitumen is necessary [4,5]. The past experience with the asphalt concrete impermeable layer indicated that the application of virgin bitumen was susceptible to bitumen aging and performance deterioration, which significantly reduced the flexibility at low temperatures and resulted in thermal cracking in the winter [6]. With respect to the PSPSs that are planned to be constructed in severe cold regions with high altitudes, the cracking resistance after long-term aging is of great importance for the bituminous materials of the impermeable layer. Compared with virgin bitumen, polymer-modified bitumen usually exhibits better aging and cracking resistance, which is believed to be vital for the long-term durability of the impermeable layer. Therefore, the application of polymer-modified bitumen is promising and has been demonstrated to have excellent performance in several PSPSs [7,8,9].

Among different types of polymer-modified bitumen, SBS-modified bitumen is most commonly used due to its excellent performance at both low and high temperatures [10,11,12]. The addition of SBS polymer into virgin bitumen can further improve the related properties to meet the critical technical requirements. The content of SBS polymer is found to be one of the key factors that affects the properties of the obtained modified bitumen. In general, different SBS contents can result in different performances as well as modification mechanisms [13,14,15,16]. When the SBS content is 4–6%, a three-dimensional polymer network structure is formed in the modified bitumen and thus obviously improves the related properties [17,18,19,20]. When the SBS content is below 4%, the bitumen phase becomes a continuous phase while the SBS phase becomes a disperse phase. In such a structure, the modification effect of the SBS polymer is due to its absorption of the oil fraction of bitumen and thus forms a polymer swelling mesophase. At high temperatures, the polymer mesophase exhibits higher strength and modulus compared with the bitumen phase, which can improve the high-temperature properties. The opposite occurs at low temperatures. The polymer mesophase exhibits lower strength and modulus compared with the bitumen phase, which can improve low-temperature flexibility. In general, the modification mechanism contributes to the absorption of the oil fractions of bitumen and thus increases the contents of resins and asphaltenes. This results in the optimization of various fractions in the bitumen and the improvement of rheological properties [21,22]. The compatibility between SBS polymer and base bitumen can be a problem due to the polymer segregation [23,24,25]. This phenomenon is likely to occur during storage and transportation at high temperatures. Polymer segregation indicates bad storage stability and thus impacts the modification function of SBS polymers [26,27,28,29]. The aging of SBS polymer modified bitumen during mixing, transportation, and paving makes the aged bitumen stiffer and more brittle and thus susceptible to fatigue damage and thermal cracking [30,31].

Many studies has been performed on the modification mechanism and the aging performance of SBS-modified bitumen with normal and high SBS polymer contents. Li et al. reported the effect of thermal aging on the microstructure and thermal stability and found that the bitumen was aged, and the polymer was degraded with an increasing degree of aging [32]. Yan et al. studied the aging behavior of highly modified bitumen with 7.5% SBS polymer content and found that the degradation of SBS polymer mainly occurred during the initial aging phase. After aging, the resilience and high-temperature properties were improved [33]. Zhang et al. prepared various SBS-modified bitumen binders with 4.5%, 6.0%, 7.5%, and 9.0% SBS polymer contents. The modification mechanism of highly modified bitumen was investigated, and it was found that SBS-modified bitumen with high polymer contents exhibited higher viscosity, better resilience, and temperature susceptibility, which leads to an improved rutting resistance of the open-graded friction course (OGFC) mixture [34]. 

SBS-modified bitumen binders with normal and high polymer contents have been widely used in the field of road engineering. Because low SBS polymer content does not exhibit an obvious improvement on bitumen properties, the application of slightly SBS-modified bitumen with low polymer content is limited in the field of road engineering. However, in the field of hydraulic engineering, the subjected stress, work environment, and construction conditions of asphalt concrete are strongly different from those in road engineering. The addition of a relatively low content (for example, 2%) of SBS polymer into base bitumen can well balance the technical indexes at low and high temperatures, and it is found to be flexible and practical to produce bitumen with different penetration degradations. This makes the application of slightly SBS-modified bitumen possible, and it has been demonstrated in several PSPSs [35]. Due to a lack of fundamental research performed on slightly SBS modified bitumen, concerns arise on the modification mechanism, long-term effectiveness, storage stability, and aging resistance. 

Slightly SBS-modified bitumen has come into use since it is not formally recognized in the field of hydraulic engineering in China. The durability thus becomes a big concern. For this reason, the goal of this study is aimed at the storage stability and aging resistance of slightly SBS modified bitumen binders that have been used in PSPSs. In total, three types of slightly SBS-modified bitumen were involved. For the purpose of comparison, a commonly used virgin bitumen and a normal SBS-modified bitumen that were used in hydraulic engineering were selected as the reference bitumens. The storage stability tests at different storage times were conducted, and the degree of polymer segregation was evaluated using the change of softening point and rheological parameters, including complex modulus and phase angle. All of the bitumen binders were subjected to laboratory simulations of short- and long-term aging. A FTIR test was performed to investigate the chemical changes before and after aging. Fundamental research on rheological properties was performed by the DSR rheological test, the stress relaxation test, and the direct tensile test. An attempt was made to establish the relationship of the aging indexes between FTIR analysis, rheological properties, and low-temperature performance. With a better insight into the aging resistance of slightly polymer-modified bitumen, an effective evaluation method for bitumen durability can be proposed. 

## 2. Materials and Methods

### 2.1. Materials

In this study, the bitumen binders consisted of Jurong SG90 bitumen and Jingbo polymer-modified bitumen (Jingbo PMB). Karamay SG70 was produced by Karamay Petrochemical Co., Ltd. (Beijing, China) of the CNPC. Because Karamay SG70 is commonly accepted and used bitumen in China, and the aging resistance and durability are highly recognized in the field of hydraulic engineering, it was selected as the reference bitumen for the purpose of comparison. Both Jingbo SG70 bitumen and Jingbo PMB binders were produced by Shandong Chambroad Petrochemicals Co., Ltd. (Binzhou, China). Both were used for the Yimeng PSPS project in Shandong Province, China. Liaohe SG90 bitumen was produced by Liaohe Petrochemical Co., Ltd. (Panjin, China) of the CNPC and was applied for the core wall of the Dunhua project in Jilin Province, China. Jurong SG90 bitumen was produced by Liaohe Petrochemical Co., Ltd. (Panjin, China) of the CNPC, and it was applied for the Jurong PSPS project in Jiangsu Province, China. 

Karamay SG70 bitumen was virgin bitumen, while Jingbo SG70 bitumen, Liaohe SG90 bitumen, and Jurong SG90 bitumen binders were not pure virgin bitumen binders but were slightly SBS-modified bitumen binders that contained a low content of SBS polymer (approximately 2%). This has been demonstrated in the previous studies using the FTIR test and DSR rheological analysis [35]. Jingbo PMB was polymer-modified bitumen with a normal content of SBS polymer (approximately 4%). More detailed information on the related properties of these five bitumen binders, as mentioned above, can be found in the literature [35].

### 2.2. Test Methods

#### 2.2.1. FTIR Spectroscopy Test

The FTIR spectroscopy test was carried out according to the similar test procedures shown in the reference [36]. Firstly, bitumen sample was dissolved to prepare a 5 wt% concentration of carbon disulfide solution. Secondly, the obtained solution was applied on a KBr slide and dried to prepare the test sample. FTIR analysis was performed within the wavelength range of 4000 cm^−1^ to 400 cm^−1^. 

In FTIR spectroscopy, the carbonyl groups (C=O) at 1700 cm^−1^ and the sulfoxides (S=O) at 1030 cm^−1^ were used to investigate the aging degree of the base bitumen. The specific absorption peaks at 966 cm^−1^ and 699 cm^−1^ were used to study the degradation of the polybutadiene (PB) and the polystyrene (PS) of SBS polymer, respectively. The carbonyl index, sulfoxide index, PB index, and PS index were calculated by the ratio of their band areas to the total spectra areas ranging from 2000 cm^−1^ to 600 cm^−1^ using Equations (1)–(4), respectively [36].

(1) $$I_{C=O}\;=\;\frac{A_{C=O}}{A}\times 100\%$$

(2) $$I_{S=O}\;=\;\frac{A_{S=O}}{A}\times 100\%$$

(3) $$I_{\mathrm{PB}}\;=\;\frac{A_{PB}}{A}\times 100\%$$

(4) $$I_{\mathrm{PS}}\;=\;\frac{A_{PS}}{A}\times 100\%$$

where:

$A_{C=O}$ is the area of carbonyl centered around 1700 cm^−1^;

$A_{S=O}$ is the area of sulfoxide centered around 1030 cm^−1^;

$A_{PB}$ is the area of polybutadiene centered around 966 cm^−1^;

$A_{PS}$ is the area of polystyrene centered around 699 cm^−1^;

A is the area of the spectral bonds between 2000–600 cm^−1^;

I_C=O_ is the carbonyl index;

I_S=O_ is the sulfoxide index;

I_PB_ is the polybutadiene (PB) index;

I_PS_ is the polystyrene (PS) index.

In order to further investigate the degree of aging, the relative change of the carbonyl index, sulfoxide index, PB index, and PS index after short- and long-term aging were defined as the short- and long-term aging index when using the unaged bitumen as the reference. An example of how to determine the short- and long-term aging index based on the carbonyl index is indicated by Equations (5) and (6), respectively. Similarly, the rest of the three specific peak indexes can also be used in the same way to determine the corresponding aging index.

(5) $${\mathrm{AI}}_{C=O-STA\;}=\frac{I_{C=O\left(STA\right)-}I_{C=O\left(UNAGED\right)}}{I_{C=O\left(UNAGED\right)}}\times 100\%$$

(6) $${\mathrm{AI}}_{C=O-LTA}\;=\;\frac{I_{C=O\left(LTA\right)-}I_{C=O\left(UNAGED\right)}}{I_{C=O\left(UNAGED\right)}}\times 100\%$$

where:

I_C=O(UNAGED)_ is the carbonyl index before aging;

I_C=O(STA)_ is the carbonyl index after short-term aging;

I_C=O(LTA)_ is the carbonyl index after long-term aging;

AI_C=O-STA_ and AI_C=O-LTA_ are the short- and long-term aging indexes based on the carbonyl index, respectively.

#### 2.2.2. Storage Stability Test

The storage stability of slightly and normal SBS polymer-modified bitumen was tested according to ASTM-D7173 [37]. After the bitumen sample was stored in an aluminum foil tube at 163 °C for 48 h, the bitumen samples in the top and bottom sections were used to conduct the softening point test. The difference in the softening point between the top and bottom sections was measured and used as an indicator of storage stability. When the difference is less than 2.5 °C, it usually indicates good storage stability for the test-modified bitumen [37].

In order to investigate the long-term storage stability, the storage time was prolonged from 2 days to 4 days and 7 days. The storage time of 4 days was used to simulate a possible scenario of extended storage time due to the temporary delay of bitumen transportation and application, while the storage time of 7 days was used to simulate the worst-case scenario of long-term storage due to the unforeseen construction interruption of asphalt mixture in the rainy season. Similarly, the softening point test was carried out to evaluate the storage stability. Furthermore, the DSR rheological test was performed to obtain insight into the change in the complex modulus and the phase angle between the top and bottom sections. To perform this, a temperature sweep test was conducted from 30 °C to 70 °C. The segregation indexes based on the change percentage of complex modulus and phase angle were determined using Equations (7) and (8), respectively.

(7) $${\mathrm{SI}}_{G_{*}}\;=\;\frac{G_{t}^{*}-G_{b}^{*}}{G_{b}^{*}}\times 100\%$$

(8) $${\mathrm{SI}}_{\delta }=\frac{\delta_{t}-\delta_{b}}{\delta_{b}}\times 100\%$$

where:

$G_{t}^{*}$ and $G_{b}^{*}$ are the complex modulus of the top and bottom sections, respectively, in Pa;

$\delta_{t}$ and $\delta_{b}$ are the phase angle of the top and bottom sections, respectively, in °, and;

SI_G*_ and SI_δ_ are the segregation indexes based on the change of the complex modulus and the phase angle, respectively.

#### 2.2.3. Laboratory Aging

In the field of hydraulic engineering in China, the thin film oven test (TFOT) is used to simulate the short-term aging of bitumen. In this study, the TFOT was applied at 163 °C on a bitumen film thickness of 3 mm for 5 h [38]. At present, there is no test method available for the long-term aging simulation in the laboratory for bitumen. According to previous research results of laboratory-accelerated aging on the asphalt mixture of the impermeable layer, it was found that the bending flexural strain at failure was strongly influenced by the aging of the mixture. Compared with specimens obtained from the field, it was found that the effect of 3.5 h of laboratory-accelerated aging was equal to the short-term aging during mixing and construction. When the time of laboratory accelerated, aging is prolonged to 15.25 h, 28.7 h, and 45.5 h, it is able to represent the aging effect of the impermeable layer after serving 14 years, 30 years, and 50 years, respectively [6]. Test results on road bitumen indicate that TFOT at 163 °C for 20 h could provide the equivalent aging effect when compared with the long-term aging simulated by using a pressure aging vessel (PAV) [39]. Siddiqui reported that four Arabian asphalt binders, after aging by a rolling thin-film oven test (RTFOT) for 340 min, exhibited similar rheological properties to those obtained by PAV aging [40]. Based on previous analysis, it was found that extending the aging time of the standard TFOT and RTFOT allowed for the simulation of long-term aging. As a rough estimate, the TFOT at 163 °C for 24 h was considered to be severe for bitumen aging and thus was selected to simulate the long-term aging of the used bitumen binders in this study. 

All five types of bitumen binders were subjected to a 5 h TFOT for short-term aging and a 24 h TFOT for long term aging. Therefore, in total, 15 types of bitumen binders were involved in this study. Table 1 lists the abbreviation of each type of bitumen binder.

#### 2.2.4. DSR Rheological Test

The rheological properties of various bitumen binders were tested by a DSR (MCR101, Anton Paar, Ostfildern, Germany). The complex modulus (G*) and phase angle (δ) were measured by the frequency sweep test mode. Different test temperatures were considered with an interval of 10 °C, in a range from −10 °C to 70 °C. The frequency sweep was set in a range of 0.1 Hz to 50 Hz. When the test temperature was below 25 °C, the 8 mm parallel plates with a 2 mm gap were used. However, when the test temperature was greater than 25 °C, the 25 mm parallel plates with a 1 mm gap were used. More information on the DSR rheological test can be found in the literature [41,42]. 

#### 2.2.5. Stress Relaxation Test

A DSR was used to evaluate the ability of stress relaxation at low temperatures under a strain-controlled method. The 8 mm parallel plates with a 2 mm gap were used for the relaxation test. Considering the difference in extreme low temperatures in southern and northern China, two test temperatures, including −10 °C and −25 °C, were selected, respectively. A constant strain was selected to ensure that the bitumen relaxation was tested in the linear viscoelastic range. For this reason, the strain level was chosen as 2.5% for −10 °C, and 0.5% for −25 °C. As the test began, the shear strain was applied instantaneously. The resultant shear loading was measured and recorded automatically for a loading period of 600 s.

The aging resistance of the different types of bitumen was evaluated using the aging index based on the initial instantaneous modulus (IIM) and modulus relaxation rate (MRR). As shown in Equations (9) and (10), the aging index based on the initial relaxation modulus was determined by the ratio of the initial relaxation modulus of aged bitumen to that of unaged bitumen. Similarly, the aging index based on modulus relaxation rate can be explained using Equations (11) and (12).

(9) $${AI(E}_{IIM\text{-}STA})=\frac{E_{IIM\text{-}STA}}{E_{IIM\text{-}0}}$$

(10) $${AI(E}_{IIM\text{-}LTA)}=\frac{E_{IIM\text{-}LTA}}{E_{IIM\text{-}0}}$$

(11) $${AI(K}_{MRR\text{-}STA)}=\frac{K_{MRR\text{-}STA}}{K_{MMR\text{-}0}}$$

(12) $${AI(K}_{MRR\text{-}LTA)}=\frac{K_{MRR\text{-}LTA}}{K_{MRR\text{-}0}}$$

where:

E_IIM-0_ is the initial instantaneous modulus of unaged bitumen binders, MPa;

E_IIM-STA_ is the initial instantaneous modulus of short-term aged bitumen binders, MPa;

E_IIM-LTA_ is the initial instantaneous modulus of long-term aged bitumen binders, MPa;

K_MRR-0_ is the modulus relaxation rate of unaged bitumen binders;

K_MRR-STA_ is the modulus relaxation rate of short-term aged bitumen binders;

K_MRR-LTA_ is the modulus relaxation rate of long-term aged bitumen binders;

AI(E_IIM-STA_) is the short-term aging index based on initial relaxation modulus;

AI(E_IIM-LTA_) is the long-term aging index based on initial relaxation modulus;

AI(K_MMR-STA_) is the short-term aging index based on modulus relaxation rate;

AI(K_MMR-LTA_) is the long-term aging index based on modulus relaxation rate.

#### 2.2.6. Direct Tensile (DT) Test

A universal tensile testing machine (SYD-0629, Beijing Hanton Science Test Instruments Co., LTD. Beijing, China) was used for the DT test. Similarly, two different temperatures, including −15 °C and −25 °C, were considered according to the extreme low temperatures in southern and northern China, respectively. The DT test was performed under a displacement-controlled mode and the applied rate was 1 mm/min. The ultimate tensile strength and the ultimate tensile strain (UTS) were determined by means of the peak force and its corresponding strain. Based on the stress-strain curve, the slope of the linear part of the initial phase was determined as the stiffness modulus (SM). Similarly, the aging indexes based on the ratio of stiffness modulus and ultimate tensile strain were defined using the following Equations (13)–(16).

(13) $$\mathrm{AI}(E_{SM\text{-}STA})=\frac{E_{SM\text{-}STA}}{E_{SM\text{-}0}}$$

(14) $$\mathrm{AI}(E_{SM\text{-}LTA})=\frac{E_{SM\text{-}LTA}}{E_{SM\text{-}0}}$$

(15) $$\mathrm{AI}({Ɛ}_{UTS\text{-}STA})=\frac{{Ɛ}_{UTS\text{-}STA}}{{Ɛ}_{UTS\text{-}0}}$$

(16) $$\mathrm{AI}({Ɛ}_{UTS\text{-}LTA})=\frac{{Ɛ}_{UTS\text{-}LTA}}{{Ɛ}_{UTS\text{-}0}}$$

where:

E_SM-0_ is the stiffness modulus of unaged bitumen binders, MPa; 

E_SM-STA_ is the stiffness modulus of short-term aged bitumen binders, MPa; 

E_SM-LTA_ is the stiffness modulus of long-term aged bitumen binders, MPa; 

ε_UTS-0_ is the ultimate tensile strain of unaged bitumen binders, %; 

ε_UTS-STA_ is the ultimate tensile strain of short-term aged bitumen binders, %; 

ε_UTS-LTA_ is the ultimate tensile strain of long-term aged bitumen binders, %;

AI(E_SM-STA_) is the short-term aging index based on the ratio of stiffness modulus;

AI(E_SM-LTA_) is the long-term aging index based on the ratio of stiffness modulus;

AI(ε_UTS-STA_) is the short-term aging index based on the ratio of ultimate tensile strain;

AI(ε_UTS-LTA_) is the long-term aging index based on the ratio of ultimate tensile strain.

## 3. Results and Discussion

### 3.1. FTIR Spectroscopy Analysis

**Figure 1:**
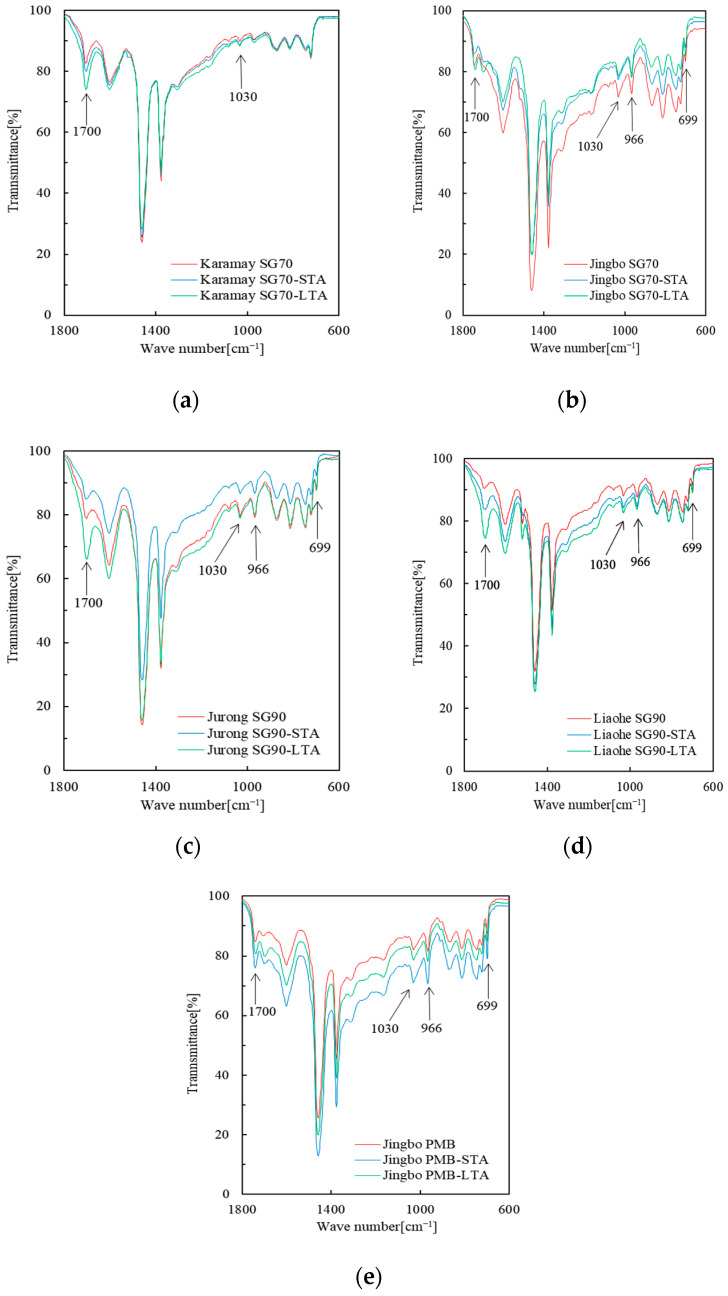
FTIR test results of various bitumen binders: (**a**) unaged, short- and long-term aged Karamay SG70; (**b**) unaged, short- and long-term aged Jingbo SG70; (**c**) unaged, short- and long-term aged Jurong SG90; (**d**) unaged, short- and long-term aged Liaohe SG90; (**e**) unaged, short- and long-term aged Jingbo PMB.

Figure 1 shows the infrared spectra of various bitumen binders. For each bitumen binder, the effect of short- and long-term aging was taken into account. The analysis of the FTIR spectroscopy results was mainly made use of the change of the four specific peaks, including the carbonyl (C=O) at 1700 cm^−1^, the sulfoxides (S=O) at 1030 cm^−1^, the polybutadiene (PB) at 966 cm^−1^, and the polystyrene (PS) at 699 cm^−1^. 

As shown in Figure 1, except for the Karamay SG70, the other four types of bitumen binders all showed obvious specific peaks at 966 cm^−1^ and 699 cm^−1^. This can be regarded as evidence of the existence of the SBS polymer. It indicated that Karamay SG70 was pure virgin bitumen and the other four bitumen binders all contained SBS polymer. In general, Jingbo PMB showed a stronger peak value compared with Jingbo SG70, Jurong SG90, and Liaohe SG90. Since Jingbo PMB was known as normal polymer modified bitumen with about 4% SBS polymer content, it implies that Jingbo SG70, Jurong SG90, and Liaohe SG90 belong to the type of slightly SBS-modified bitumen. These analysis results were in agreement with the previous studies based on the FTIR test and the DSR rheological test [35]. 

Table 2 presents the aging index based on the carbonyl index, sulfoxide index, PB index, and PS index. It should be noted that all of these aging indexes used the corresponding unaged bitumen binder as a reference. Therefore, a higher aging index indicated a more susceptible aging resistance. As listed in Table 2, after short- and long-term aging, the aging indexes based on the carbonyl and sulfoxide peaks increased independently of the type of bitumen. This indicated the aging of the base bitumen. Short-term aging could result in a sulfoxide aging index within 0.2–8.3%, while the carbonyl aging index ranged from 15.4–72.7%. After long-term aging, these two indexes were within 4.7–20.5% and 59.3–840.5%, respectively. This indicated that the carbonyl index was more sensitive to bitumen aging compared with the sulfoxide index [43,44]. Long-term aging had a more profound effect than short-term aging. In this point, long-term aging should be used for the bitumen ranking in terms of long-term durability. 

With respect to bitumen aging resistance, different aging indexes could lead to different results. When the sulfoxide aging index was used, it was found that Jurong SG90, Liaohe SG90, and Jingbo PMB tended to have excellent aging resistance. Karamay SG70 and Jingbo SG70 had a relatively high sulfoxide aging index compared to the other three bitumen binders, as mentioned above. When the carbonyl aging index was used, Jingbo PMB and Karamay SG70 showed excellent aging resistance, while Jingbo SG70, Jurong SG90, and Liaohe SG90 showed high aging indexes regardless of short- and long-term aging. In general, Jingbo PMB showed better aging resistance compared with Jingbo SG70, which indicated that increasing the SBS polymer content improved the aging resistance in the case of the same base bitumen. When using Karamay SG70 as a reference, the three types of slightly SBS-modified bitumen did not have a profound improvement on aging resistance. 

With respect to polymer degradation, both the PB index and the PS index could give a consistent result. Jingbo PMB showed the best resistance of polymer degradation, while Jingbo SG70, Jurong SG90, and Liaohe SG90 show a similar resistance of polymer degradation regardless of short- and long-term aging. It seemed that the aging indexes based on the PB index, and the PS index were strongly related to the type of bitumen binder, especially for the content of SBS polymer. The slightly SBS-modified bitumen binders, including Jingbo SG70, Jurong SG90, and Liaohe SG90 tended to have an aging index around −10% for short-term aging, while Jingbo PMB had a corresponding aging index of −0.7% to −3.6% based on the PB index and the PS index. After long-term aging, the aging index was found to be in the order of −15% to −20% for the slightly SBS-modified bitumen binders, and only −6.2% and −1.4% for Jingbo PMB. The above analysis indicated that slightly SBS-modified bitumen binders were more susceptible to polymer degradation compared with normal Jingbo PMB. This may reflect the difference in the related rheological properties. Therefore, it is foreseen that short- and long-term aging may have a more disadvantageous influence on slightly SBS-modified bitumen compared with the normal SBS modified bitumen. When considering the sensitivity between the PB index and the PS index, it is found that the aging index based on the PS index varied relatively little for Jingbo PMB, while the PB index gave a more reasonable result. Futhermore, according to the mechanism of degradation of SBS polymer, it was reported that the aging of PB is easier than that of PS. References [45,46] demonstrated that the PB index was more sensitive to the PS index and recommended the PB index for the degradation of the SBS polymer.

By combining aging indexes obtained from the FTIR indexes, it was found that the aging of base bitumen and the degradation of SBS polymer occurred during short-term and long-term aging. These two phenomena could play an important role in the related properties of aged bitumen binder. In general, Karamay SG70 was a base bitumen, so the aging behavior was directly related to the aging of the base bitumen. Because Jingbo SG70 bitumen, Liaohe SG90 bitumen, and Jurong SG90 bitumen were not pure virgin bitumen but slightly SBS-modified bitumen containing a small amount of SBS polymer (approximately 2%), their aging behavior was due to a combination of the aging of the base bitumen and the degradation of the SBS polymer. For slightly modified bitumen, the aging of the base bitumen may be dominant compared with the degradation of the SBS polymer. With respect to Jingbo PMB, which is a polymer-modified bitumen with a normal content of SBS polymer (approximately 4%), both the aging of base bitumen and the degradation of SBS polymer may be important for the aging behavior. In general, Jingbo PMB exhibited excellent performance after aging, while the three types of slightly SBS modified bitumen exhibited more serious aging of base bitumen together with a higher degree of polymer degradation.

### 3.2. Storage Stability Test Results

Table 3 shows the storage stability test results based on the difference in the softening point. For the purpose of comparison, the softening point of the unaged bitumen binder was also listed. As indicated in Table 3, the softening point tended to increase after storing at 163 °C for 48 h regardless of the top and bottom sections. This can be explained by the aging effect during the storage stability test. The top section showed a relatively high softening point compared to the bottom section, which indicates the segregation of the SBS polymer. However, the difference in the softening point between the top and bottom sections was smaller than 1 °C. When using Jingbo PMB as a reference, it could be concluded that the three slightly SBS-modified bitumen binders had excellent storage stability.

**Figure 2:**
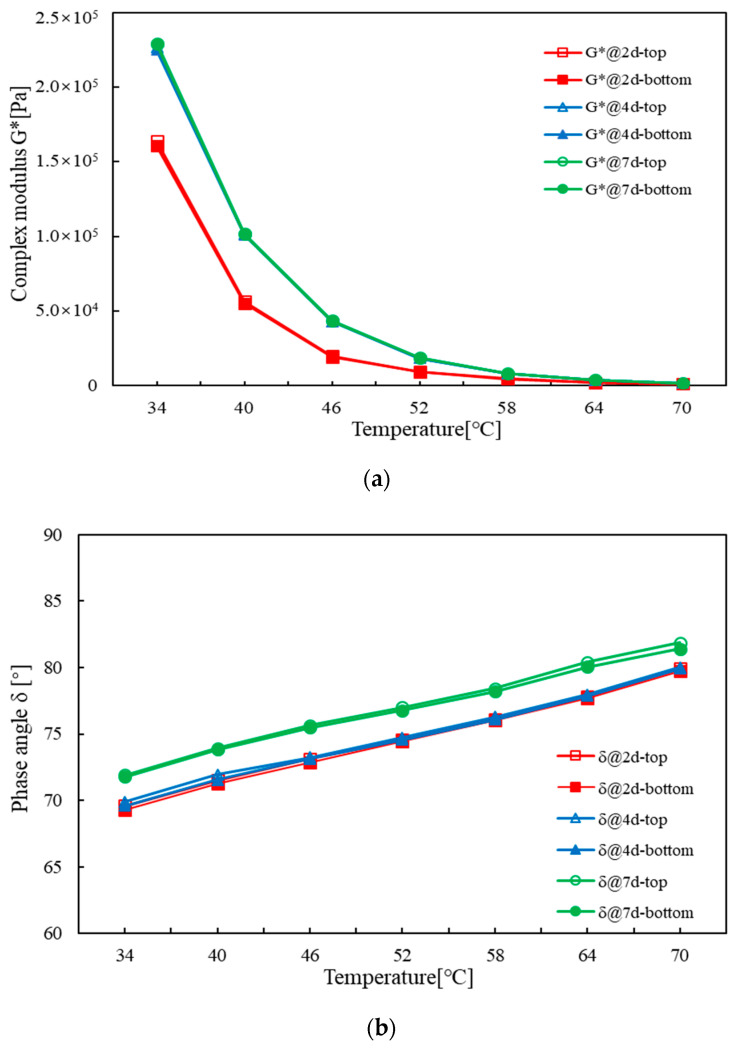

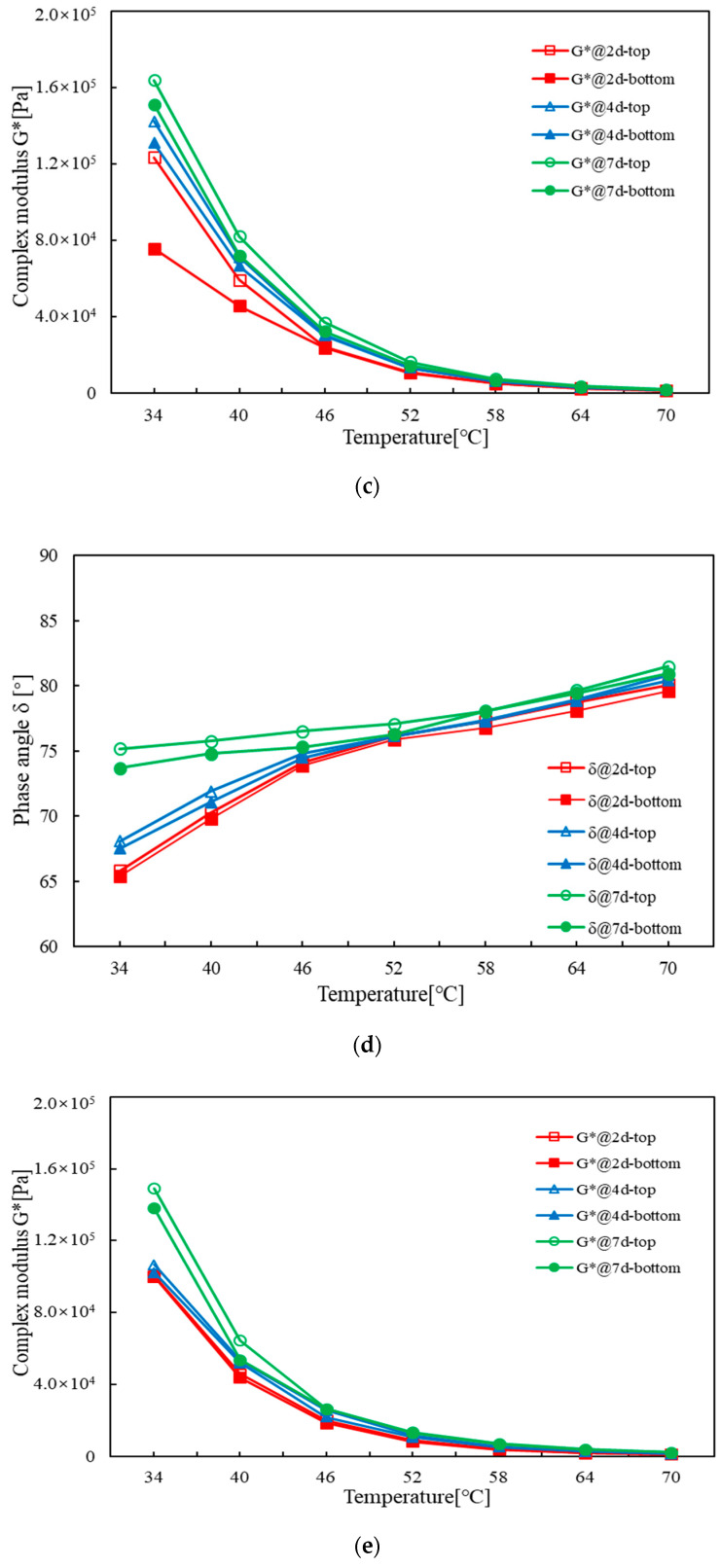

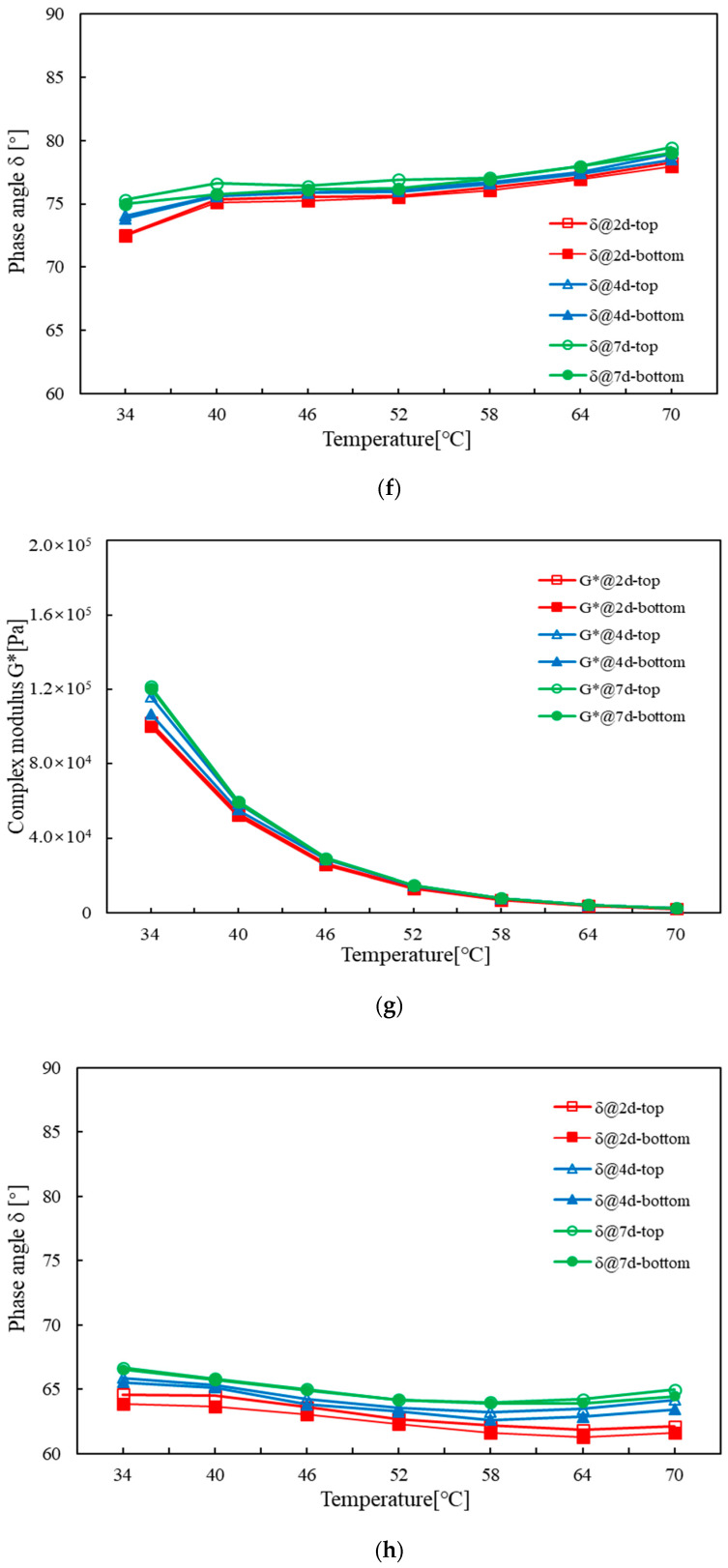
Rheological properties of various bitumen binders at different storage times: (**a**) complex modulus of Jingbo SG70 bitumen; (**b**) phase angle of Jingbo SG70 bitumen; (**c**) complex modulus of Jurong SG90 bitumen; (**d**) phase angle of Jurong SG90 bitumen; (**e**) complex modulus of Liaohe SG90 bitumen; (**f**) phase angle of Liaohe SG90 bitumen; (**g**) complex modulus of Jingbo PMB bitumen; (**h**) phase angle of Jingbo PMB bitumen.

In order to investigate the effect of storage time on the rheological properties, the softening point test was replaced by the DSR temperature sweep test. Firstly, the storage stability test was carried out on various bitumen binders for 2, 4, and 7 days of storage time at 163 °C, respectively. After that, the bitumen samples at the top and bottom sections were respectively applied for the DSR rheological test. Figure 2 presents the temperature sweep test results at different storage times in terms of the complex modulus and the phase angle. The segregation indexes based on the ratio of complex modulus and phase angle are listed in Table 4 and Table 5, respectively.

As indicated in Figure 2, the storage time had an obvious influence on the rheological properties of the tested bitumen binders. In general, an increasing storage time resulted in an increased complex modulus, but a decreased phase angle. The difference in complex modulus between the top and bottom sections seemed to be limited. A similar result was also seen on phase angle. The segregation indexes listed in Table 4 and Table 5 further demonstrated the stability of the rheological properties after long-term storage. 

With respect to the complex modulus segregation index as listed in Table 4, it ranged from 0.128% to 14.960% depending on the test temperature, the type of bitumen, and the storage time. The type of bitumen had a significant influence on the segregation index. After 7 days of storage, Jurong SG90 had the highest segregation index of 14.960%, while Jingbo SG70 had the lowest one of 3.238%. The segregation index also exhibited temperature dependency, and it seemed to increase as temperature increased. Therefore, it is recommended to use the segregation index at a relatively high temperature, for example 64 °C, for the evaluating indicator. The segregation index increased with increased storage time. Four days of storage time seemed to be the critical time for the complex modulus segregation index. For long-term storage stability, the segregation index at 64 °C for 4 days should be controlled to no larger than 10% based on the results of this study. When using Jingbo PMB as a reference, Jingbo SG70 and Liaohe SG90 exhibited an equivalent rheological stability, while Jurong SG90 tended to have a susceptible rheological stability.

With respect to the phase angle segregation index listed in Table 4, it remained relatively constant, within 2%, regardless of test temperature, type of bitumen, and storage time. This indicated that the phase angle segregation index was not sensitive to the polymer segregation when compared to the complex modulus. In this case, the phase angle did not seem to be a good evaluating indicator for rheological stability. 

By combining the test results obtained from softening point and DSR rheological test, it can be concluded that the slightly SBS-modified bitumen binders had an acceptable storage stability. Even at a disadvantagous long-term storage period of 7 days, the rheological properties were able to remain fairly constant.

### 3.3. Rheological Master Curve

**Figure 3:**
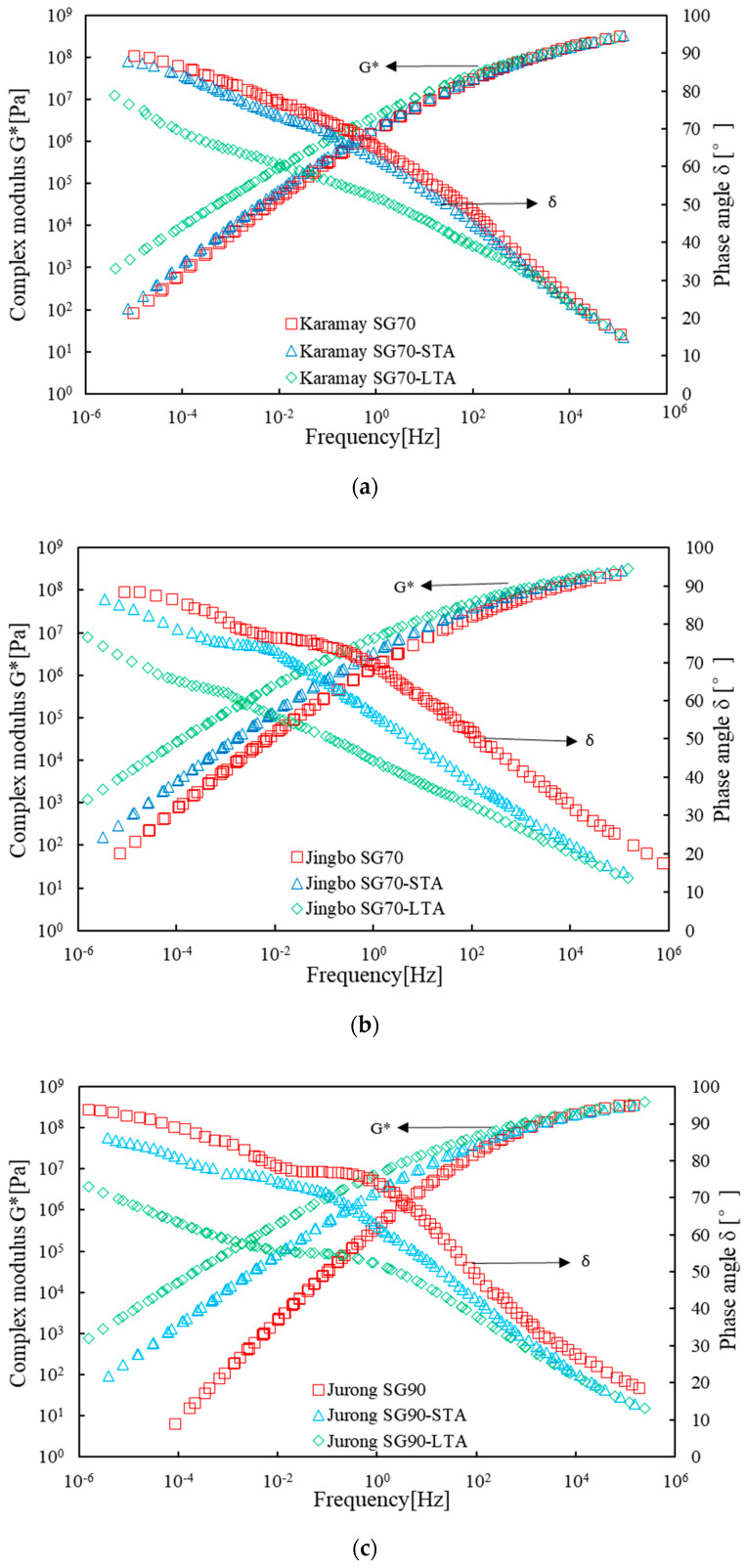

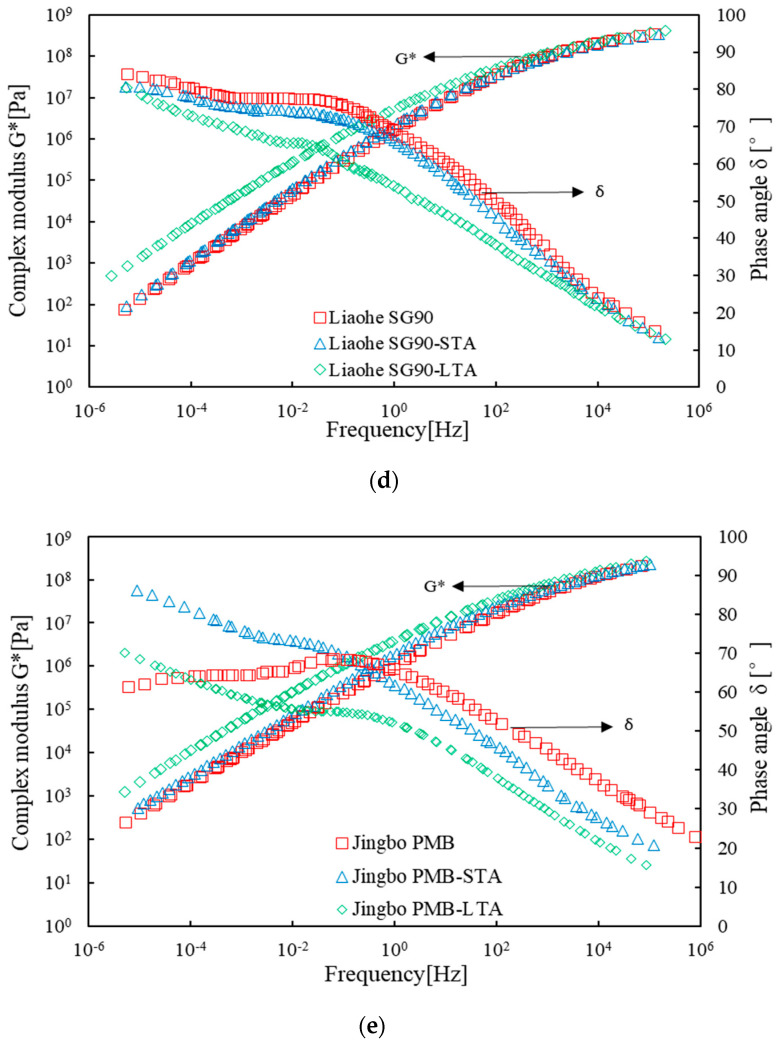
Master curves of complex modulus and phase angle of unaged, short- and long-term aged bitumen binders: (**a**) Karamay SG70; (**b**) Jingbo SG70; (**c**) Jurong SG90; (**d**) Liaohe SG90; (**e**) Jingbo PMB.

Figure 3 presents the rheological master curves for various bitumen binders. All of the master curves were constructed based on the test results obtained from the frequency sweep testing. A reference temperature of 20 °C was chosen, and the frequency sweep test data at other temperatures were shifted horizontally to the reference temperature. Finally, a smooth master curve over a wide range of frequencies was established. More information on how to construct the master curve can be found in [35].

As indicated in Figure 3, aging resulted in an increased complex modulus but decreased the phase angle over a wide range of frequencies. Long-term aging had a more profound influence on the complex modulus and the phase angle when compared to short-term aging. As indicated by the FTIR test results, Karamay SG70 was a pure virgin bitumen, while the other four types of bitumen binders were found to contain SBS polymer. This result was also well demonstrated by the master curve of the phase angle. Compared with Karamay SG70, there was a remarkable plateau of the phase angle for Jingbo PMB, Jingbo SG70, Liaohe SG90, and Jurong SG90. Previous studies demonstrated that the plateau of the phase angle master curve strongly indicated the existence of the polymer [35,36]. Because the degree of change of the phase angle plateau was usually related to the polymer content, it was deduced that Jingbo SG70, Jurong SG90, and Liaohe SG90 contained a relatively small amount of SBS polymer when compared to Jingbo PMB, which usually had a SBS polymer content of around 4%. After aging, the plateau of the phase angle tended to change, and this change was more noticeable after long-term aging. This indicated the degradation of the SBS polymer as well as damage of the polymer network structure [28,32]. This result was in agreement with the PB index, and the PS index obtained from the FTIR analysis. 

The effect of short- and long-term aging on the master curve was well distinguished in the range of low frequencies when compared with the range of high frequencies. Furthermore, after short- and long-term aging, the change trends of complex modulus master curves were more consistent when compared with phase angle master curves. The complex modulus master curve after short-term aging deviated from the one of unaged bitumen. Further deviations could be seen after long-term aging. For this reason, the degree of deviation of the complex modulus master curve can be regarded as the rheological aging index. 

**Figure 4:**
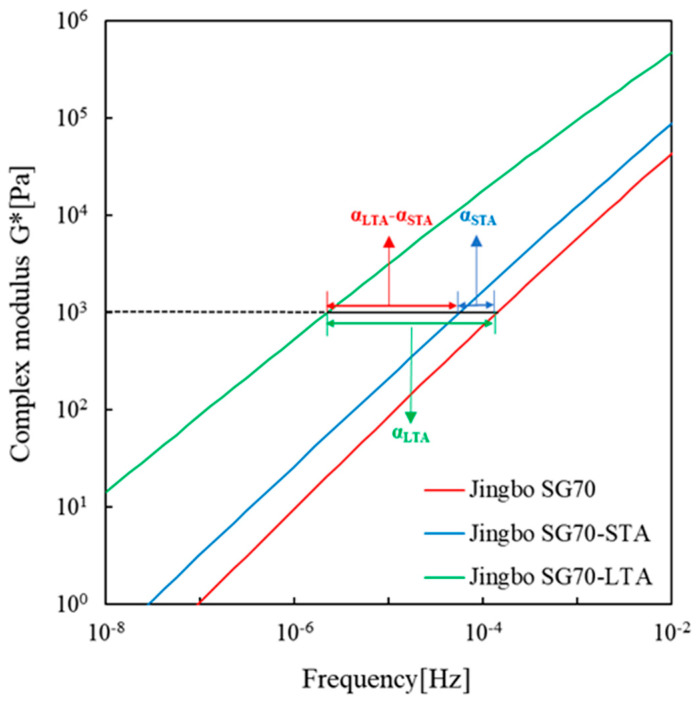
Schematic diagram of the horizontal shift factor with a reference complex modulus at 1000 Pa.

**Figure 5:**
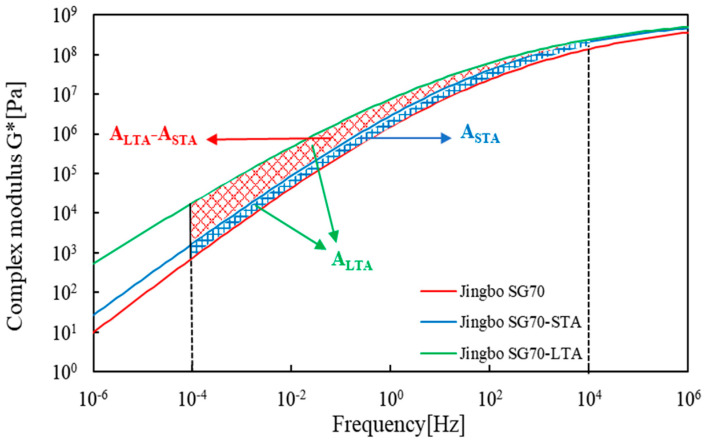
Schematic diagram of the deviation areas of Jingbo SG70 bitumen.

The degree of deviation of the complex modulus master curve can be explained by the horizontal shift factor, as indicated in Figure 4, and the deviation area, as illustrated in Figure 5. The horizontal shift factor and the deviation area were determined based on the log-log scale plot of the complex modulus master curve. For the horizontal shift factor, the reference modulus needed to be selected and a complex modulus of 1000 Pa was used in this study. The corresponding frequencies equal to the reference modulus were determined for unaged, short-, and long-term aged bitumen, respectively. The difference in logarithmic frequency between short-term aged and unaged bitumen is defined as the horizontal shift factor between short-term aged and unaged bitumen. Similarly, one can obtain the horizontal shift factor between long-term aged bitumen and unaged bitumen as well as between long-term aged bitumen and short-term aged bitumen. By this method, the shift factors $\alpha_{STA}$ and $\alpha_{LTA}$ were determined and are listed in Table 6.

The deviation area was defined as the enclosed area between the master curve of unaged bitumen and the corresponding aged bitumen within the frequency region ranging from 10^−4^ Hz to 10^4^ Hz. By doing this, the deviation areas for short- and long-term aging, that is, $A_{STA}$ and $A_{LTA}$ were determined and listed in Table 6.

As indicated in Table 6, the rheological aging index can be well explained by the horizontal shift factor and the deviation area after short- and long-term aging. For short-term aging, the horizontal shift factor $\alpha_{STA}$ ranged from 0.10 to 1.97. Jingbo PMB and Liaohe SG90 seemed to be insensitive to short-term aging, while Jurong SG90 was the most susceptible. After long-term aging, the horizontal shift factor $\alpha_{LTA}$ increased and varied from 1.01 to 3.37. Except for Jurong SG90, the other four types of bitumen binders seemed to have a close shift factor within one to two. The value of $\alpha_{LTA}-\alpha_{STA}$, which indicated the difference between short- and long-term aging, showed a similar result for the five types of bitumen binders. 

With respect to the deviation area, $A_{STA}$ ranged from 0.60 to 7.68 for short-term aging. Jingbo PMB and Liaohe SG90 seemed to be insensitive to short-term aging, while Jurong SG90 was the most susceptible. After long-term aging, the deviation area $A_{STA}$ obviously increased and varied from 3.48 to 11.42. Jurong SG90 had a deviation area in an order of 10, while the other three types of bitumen binders seemed to have a close deviation area of 3 to 6. The value of $A_{LTA}-A_{STA}$ showed a close result except for Karamay SG70.

The analysis above showed that the horizontal shift factor and the deviation area can generally give a consistent ranking for aging resistance for the five types of bitumen binders. This indicated that the proposed rheological aging index can be used for bitumen ranking. In general, Jingbo PMB and Liaohe SG90 had excellent aging resistance, which was comparable to that of Karamay SG70. Jingbo SG70 behaved slightly worse than Karamay SG70. Jurong SG90 performed worst regardless of short- and long-term aging.

### 3.4. Stress Relaxation Test Results

**Figure 6:**
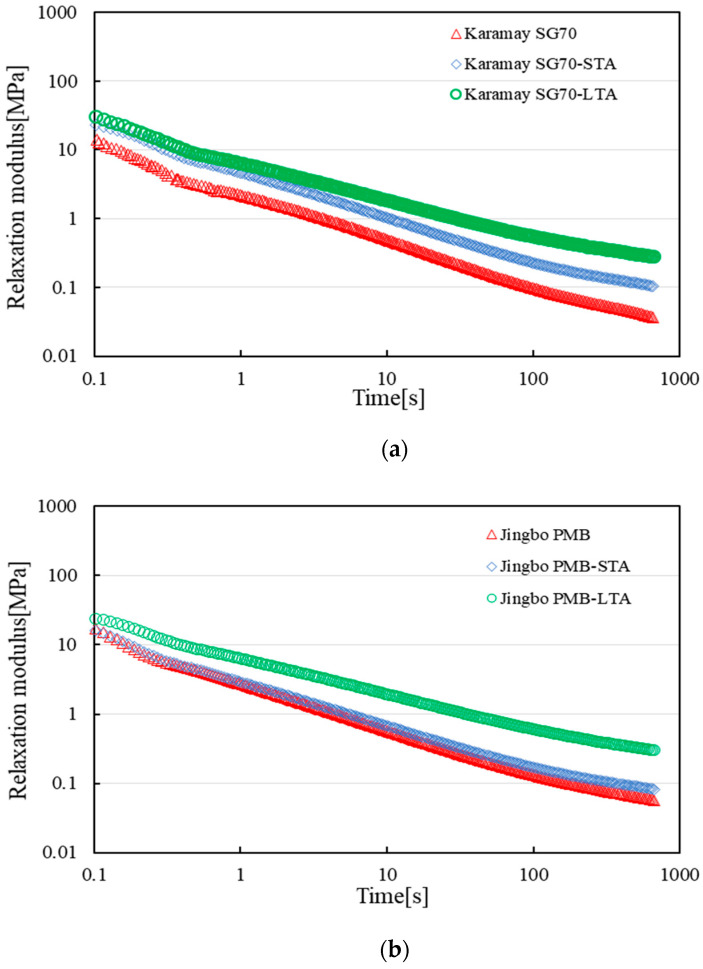
Development of a relaxation modulus over time for two types of bitumen binders at −10 °C: (**a**) Karamay SG70 bitumen; (**b**) Jingbo PMB bitumen.

Figure 6 presents the relaxation modulus development over time for Karamay SG70 and Jingbo PMB at −10 °C. Similar results were obtained for the other three types of bitumen binders. For the sake of simplicity, only the test results of Karamay SG70 and Jingbo PMB were illustrated as examples. The two selected types of bitumen binders represented the virgin bitumen and the polymer-modified bitumen, respectively. Both of these two types of bitumen binders exhibited similar modulus relaxation behaviors on the log-log scale plot. Within 600 s, the stress relaxed very fast, which led to the relaxation modulus reducing by several orders of degree. After short-term aging, the modulus relaxation rate tended to decrease, and the final residual modulus increased. Long-term aging further decreased the modulus relaxation rate and increased the final residual modulus. As indicated in Figure 6, the modulus relaxation over time followed a linear relationship in the log-log scale plot. Therefore, Equation (17) was used to fit the data obtained from the stress relaxation test at −10 °C. The fitting results were listed in Table 7.

(17) $$logE\left(t\right)=-Klog\left(t\right)+logE_{0}$$

where:

E(t) is the relaxation modulus, MPa;

E_0_ is the initial instantaneous modulus, MPa; 

t is the relaxation time, s;

K is the slope of the linear relationship on the log-log scale plot, which stands for the modulus relaxation rate.

**Table 7:** Fitting results for the relaxation test at −10 °C.

Type of Bitumen	E_0_ [MPa]	E@_0.1s_ [MPa]	E@_1s_ [MPa]	E@_10s_ [MPa]	E@_600s_ [MPa]	K	R^2^
Karamay SG70	199.17	15.42	2.18	0.49	0.04	0.678	0.980
Karamay SG70-STA	218.63	22.52	4.66	1.02	0.11	0.646	0.991
Karamay SG70-LTA	221.63	30.65	6.44	1.84	0.29	0.546	0.984
Jingbo SG70	189.52	19.39	3.90	0.78	0.04	0.732	0.997
Jingbo SG70-STA	195.64	13.60	3.26	0.79	0.09	0.613	0.990
Jingbo SG70-LTA	231.11	14.86	4.29	1.53	0.27	0.476	0.978
Jurong SG90	241.67	16.36	5.15	1.65	0.15	0.768	0.989
Jurong SG90-STA	274.72	79.70	10.89	1.84	0.17	0.739	0.988
Jurong SG90-LTA	352.19	87.25	15.95	1.92	0.21	0.531	0.987
Liaohe SG90	261.49	32.03	6.16	1.21	0.08	0.711	0.983
Liaohe SG90-STA	283.48	40.36	7.38	1.58	0.11	0.703	0.987
Liaohe SG90-LTA	363.45	49.58	9.56	1.64	0.15	0.689	0.990
Jingbo PMB	133.01	16.89	2.73	0.56	0.06	0.659	0.989
Jingbo PMB-STA	146.4	16.04	2.94	0.67	0.08	0.618	0.987
Jingbo PMB-LTA	184.16	24.12	6.36	1.93	0.31	0.519	0.988

As shown in Table 7, the initial instantaneous modulus E_0_ gradually increased after short- and long-term aging, while the value of K decreased. This indicated that all of the tested bitumen binders became stiffer after aging and lost some stress relaxation ability. In general, a low value of E_0_, together with a high value of K, is desired for a good bitumen binder in terms of stress relaxation at low temperatures. Among these five types of bitumen binders, Jingbo PMB had the lowest value of E_0_, which may be due to the modification function of the SBS polymer. Liaohe SG90 and Jurong SG90 tended to have the highest value of E_0_ after long-term aging. 

With respect to the value of K, it ranged from 0.531 to 0.768 regardless of type of bitumen and aging. This indicated that all tested bitumen binders exhibited a similar modulus relaxation rate. For example, the initial instantaneous modulus of unaged Karamay SG70 was 199.17 MPa. After it was relaxed for 0.1 s, 1 s, 10 s, and 600 s, the relaxation modulus became 15.42 MPa, 2.18 MPa, 0.49 MPa, and 0.04 MPa, respectively. After a 600 s relaxation, the relaxation modulus remained in the order of 0.1 MPa for all bitumen binders. A low final residual relaxation modulus also indicated a low risk of thermal cracking. 

Table 8 presents the fitting results for the relaxation test before and after aging at −25 °C. Compared with the data at −10 °C that is listed in Table 8, it was observed that the value of E_0_ increased as the test temperature reduced to −25 °C. For all bitumen binders, the value of E_0_ ranged from 321.84 MPa to 660.46 MPa and tended to gradually increase after short-term and long-term aging. This indicated that the bitumen became stiffer and more brittle at a lower temperature and after extended aging. Among all of the bitumen binders, Jurong SG90 had the highest value of E_0_, while Jingbo PMB had the lowest value of E_0_ after long-term aging.

With respect to the value of K, it was found to be within a range of 0.347 to 0.548 and obviously decreased compared with the one at −10 °C. This indicated a reduced modulus relaxation rate as the test temperature was further decreased from −10 °C to −25 °C. Among all of the bitumen binders, Jurong SG90 had the lowest value of K, while Liaohe SG90 had the highest value of K after long-term aging.

It should be noted that a high value of E_0_ together with a low value of K usually implies a high risk for bitumen thermal cracking. The data listed in Table 7 and Table 8 indicated that the threshold level for E_0_ and K seemed to be 300 MPa and 0.50, respectively. When using Karamay SG70 as the reference, it can be observed that slightly SBS modified bitumen binders, including Jingbo SG70, Jurong SG90, and Liaohe SG90, behaved similarly to Karamay SG70 but not Jingbo PMB for the low-temperature relaxation test. The contribution of the slight SBS modification was not so obvious when compared with Jingbo PMB. In general, the value of E_0_ was more sensitive to the type of bitumen and aging, while the change of K was relatively limited. The combination of E_0_ and K is believed to be decisive for the final relaxation residual modulus and stress, thus affecting the thermal cracking resistance. 

Table 9 presents the ratios of initial instantaneous modulus E_0_ and the modulus relaxation rate K, using the unaged bitumen binder as the reference. As shown in Equations (9)–(12), the aging indexes including AI(E_IIM-STA_), AI(E_IIM-LTA_), AI(K_MRR-STA_), and AI(K_MRR-LTA_) were defined as the ratio of E_0_ and K after short- and long-term aging, respectively. At −10 °C, the value of AI(E_IIM-STA_) varied from 1.032 to 1.137, while it ranged from 1.018 to 1.053 at −25 °C, which indicated a limited change in E_0_ after short-term aging. After long-term aging, the value of AI(E_IIM-LTA_) ranged between 1.113 and 1.390 at −10 °C, while it was within 1.035 to 1.629 at −25 °C. It indicated that long-term aging could be a good method to rank the durability of bitumen. 

With respect to the aging index based on the modulus relaxation rate, the value of AI(K_MRR-STA_) ranged from 0.837 to 0.989 at −10 °C after short-term aging, while it varied from 0.799 to 0.931 at −25 °C. After long-term aging, the value of AI(K_MRR-LTA_) was between 0.650 and 0.969 at −10 °C, while it varied from 0.717 to 0.858 at −25 °C. In general, short-term aging could lead to a loss of 5–10% of the modulus relaxation rate, while the loss could increase to 20–30% after long-term aging. 

The above analysis indicated that the effects of short- and long-term aging on stress relaxation can be well characterized using the aging index determined from the change in E_0_ and K. Both aging indexes seemed to be sensitive to the type of bitumen and aging, as well as the test temperature. The reduction of relaxation ability can be explained by the increase of the modulus together with the reduction of the modulus relaxation rate. When using Karamay SG70 as the reference, the contribution of slight modification seemed to be limited to improving the stress relaxation ability before and after aging. This was especially true at lower temperatures. 

### 3.5. Direct Tensile (DT) Test Results

**Figure 7:**
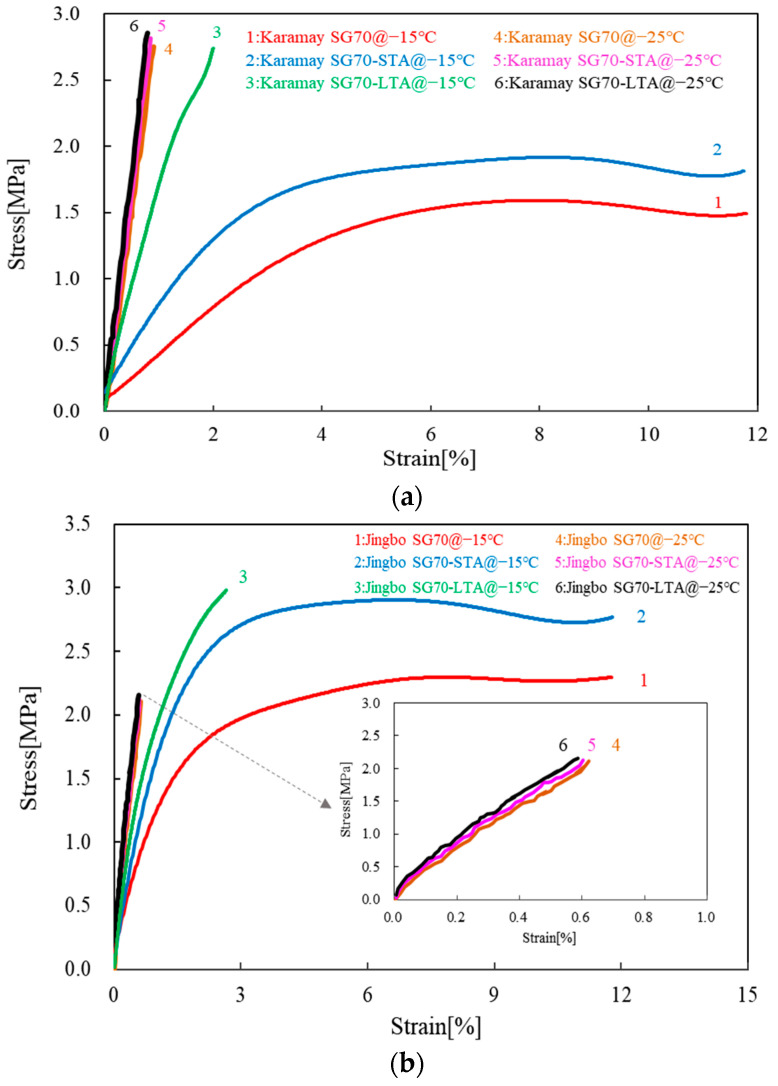

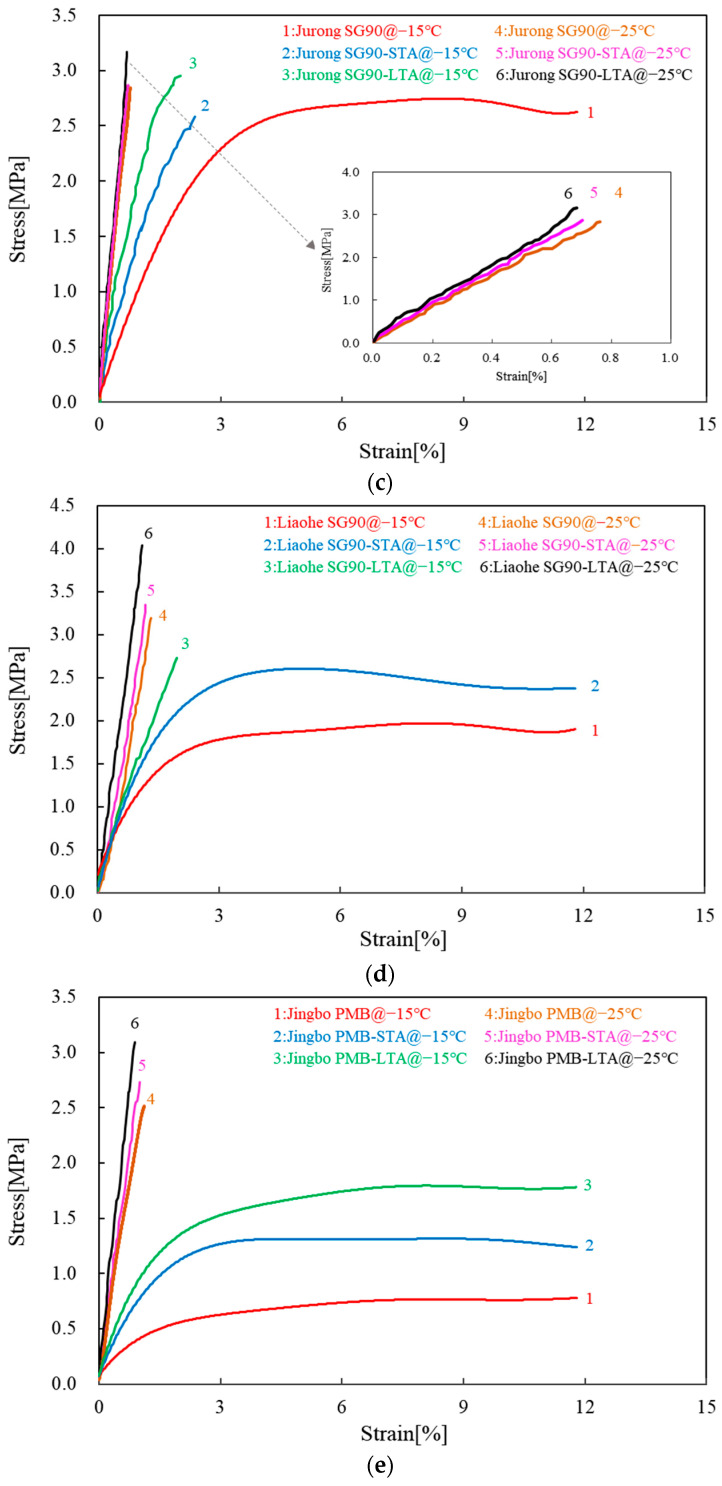
Tensile stress-strain curves of unaged, short-, and long-term aged bitumen binders: (**a**) Karamay SG70 bitumen; (**b**) Jingbo SG70 bitumen; (**c**) Jurong SG90 bitumen; (**d**) Liaohe SG90 bitumen; (**e**) Jingbo PMB bitumen.

Figure 7 presents the stress-strain curves obtained from the direct tensile tests on different bitumen binders. For each type of bitumen, the effects of short- and long-term aging were involved, as well as the effect of temperature, for instance, at −15 °C and −25 °C. Table 10 and Table 11 give a summary of the failure mode, ultimate tensile strength, ultimate tensile strain, and stiffness modulus of the direct tensile test results at −15 °C and −25 °C, respectively. 

As indicated in Figure 7, the failure mode was strongly dependent on the type of bitumen, the test temperature, as well as the effect of aging. For example, Karamay SG70 showed ductile failure for unaged and short-term aged bitumen binders at −15 °C, while it turned brittle after long-term aging. As the test temperature was decreased to −25 °C, the unaged, short-, and long-term aged bitumen binders all exhibited brittle failure. Similar results were also observed on Jingbo SG70. The main difference was seen in the ultimate tensile strength at −25 °C, which became smaller compared with those of the Karamay SG70. With respect to Jurong SG90, it tended to be more susceptible to aging. Both short- and long-term aged bitumen binders exhibited brittle failure at −15 °C. Liaohe SG90 behaved similarly to Karamay SG70, and only unaged and short-term aged bitumen binders had ductile failure. Jingbo PMB showed a large difference in the failure mode between −15 °C and −25 °C. The tensile failure was ductile at −15 °C while it turned brittle at −25 °C, which indicated that all of the bitumen binders became very brittle regardless of the type of bitumen and aging. At −15 °C, Karamay SG70, Jingbo SG70, and Liaohe SG90 were lost to ductility after long-term aging, while Jurong SG90 was lost to ductility after short-term aging. 

As indicated in Table 10, the ultimate tensile strength ranged from 0.80 MPa to 2.99 MPa for all of the tested bitumen binders. Among these bitumen binders, Jingbo PMB had the lowest strength, while Jingbo SG70 and Jurong SG90 tended to have the highest strength. Short- and long-term aging tended to increase the ultimate tensile strength. The data listed in Table 11 indicated that the ultimate tensile strength increased as the test temperature decreased. Similar results were also found for the stiffness modulus. Both extended aging and reduced the test temperature and led to the increase of the stiffness modulus. In general, the effect of short-term aging on the stiffness modulus was relatively small, while an obvious increase was observed after long-term aging. This may have a direct influence on the ultimate tensile strain due to the increase of brittleness.

The ultimate tensile strain is the key result obtained from the direct tensile test at low temperatures. It is a commonly used indicator for the flexibility and the resistance of thermal cracking. As indicated in Table 10, the ultimate tensile strain ranged from 1.71% to 8.41% for all of the tested bitumen binders. Among these bitumen binders, Jingbo PMB had the highest tensile strain, while Liaohe SG90 and Jurong SG90 tended to have the lowest tensile strain. Short- and long-term aging tended to reduce the ultimate tensile strain. When compared to the data listed in Table 11, it was found that the ultimate tensile strain was dramatically reduced to the same level of around 1% as the test temperature decreased to −25 °C. At −25 °C, extended aging only led to a limited reduction of ultimate tensile strain. In general, the effect of short-term aging on ultimate tensile strain was relatively small, while an obvious reduction was observed after long-term aging at −15 °C except for Jingbo PMB. The advantage of Jingbo PMB became relatively limited at −25 °C when compared with the other four types of bitumen binders. 

Based on the knowledge of asphalt pavement, the bitumen binder should have a tensile strain greater than 1% together with a stiffness modulus below 300 MPa to resist the thermal cracking at extremely low temperatures [47]. According to this rule, it can be concluded that all of the bitumen binders had a good thermal cracking resistance at −15 °C even after short-term and long-term aging. However, at −25 °C, only Liaohe SG90 and Jingbo PMB could meet the above requirements on the tensile strain and stiffness modulus under unaging and short-term aging conditions. After long-term aging, the stiffness modulus of Liaohe SG90 and Jingbo PMB were 364.58 MPa and 350.51 MPa, respectively. Furthermore, Jingbo PMB also had a tensile strain of 0.88, which was lower than 1% after long-term aging. Combined with the failure mode and the ultimate tensile strain listed in Table 10 and Table 11, it can be found that the brittle failure was likely to occur at the ultimate tensile strain smaller than 2.5%. Therefore, it can be concluded that the requirement for a tensile strain greater than 1% together with a stiffness modulus below 300 MPa is critical and could be used as an evaluating indicator for thermal cracking resistance. The minimum test temperature meeting this requirement can be regarded as the brittle cracking temperature. 

Table 12 presents the aging indexes determined by the ratio of the stiffness modulus and the ultimate tensile strain using the unaged bitumen binder as the reference. The aging indexes AI(E_SM-STA_), AI(E_SM-LTA_), AI(ε_UTS-STA_), and AI(ε_UTS-LTA_) were defined as the ratios of stiffness modulus and ultimate tensile strain after short- and long-term aging, respectively. At −15 °C, the value of AI(E_SM-STA_) varied from 1.208 to 3.281, while it ranged from 1.041 to 1.196 at −25 °C. A limited change of E_0_ was observed after short-term aging for most of the bitumen binders. After long-term aging, the value of AI(E_SM-LTA_) ranged between 2.289 and 6.000 at −15 °C, while it was within 1.084 to 1.559 at −25 °C. This indicated that the temperature reduction resulted in a decreased aging index based on stiffness modulus. The reason may be due to the relatively small fraction of hardening effect of aging when compared with the high stiffness modulus at extremely low temperatures.

With respect to the aging index based on the ultimate tensile strain, the value of AI(ε_UTS-STA_) ranged from 0.281 to 0.999 at −15 °C after short-term aging, while it varied from 0.908 to 0.954 at −25 °C. After long-term aging, the value of AI(ε_UTS-LTA_) was between 0.204 and 0.988 at −15 °C, while it varied from 0.786 to 0.908 at −25 °C. In general, short-term aging could lead to a loss of 5−10% in ultimate tensile strain for most asphalt binders, while the loss could increase to 60–80% after long-term aging at −15 °C. Because the ultimate tensile strain was already very small, long-term aging could only have a limited effect on AI(ε_UTS-LTA_) and thus the loss of AI(ε_UTS-LTA_) was within 10−20%.

The above analysis indicated that the effects of short- and long-term aging on the direct tensile test at low temperatures can be well characterized using the ultimate tensile strain and stiffness modulus. In general, bitumen with a high ultimate tensile strain together with low stiffness modulus is desired. The aging index, determined from the relative change of the ultimate tensile strain and stiffness modulus, is well reflected in the aging resistance. The increase in the stiffness modulus and the loss of ultimate tensile strain can be used to evaluate the susceptibility of bitumen aging. In general, Jingbo PMB and slightly modified bitumen had comparable performance to Karamay SG70 even after short- and long-term aging. 

### 3.6. Correlation Analysis

Table 13 shows the relationship between the FTIR aging index and the rheological aging index. The FTIR aging index was determined by linear regression using the rheological aging index. The FTIR aging index made use of AI_C=O-STA_, AI_C=O-LTA_, AI_PB-STA_, and AI_PB-LTA_, while the rheological aging index, including $\alpha_{STA}$, $\alpha_{LTA}$, A_STA_, and A_LTA_ was used. As indicated by the correlation coefficient, R^2^, in Table 13, there was not a good relationship between the FTIR aging index and the rheological aging index. Among these relationships, the highest correlation coefficient was found for the relationship between $\alpha_{LTA}$ and AI_PB-LTA_, with a correlation coefficient of 0.3309. It indicated that the chemical and rheological changes of bitumen after aging were not always consistent.

Table 14 shows the relationship between the rheological aging index and the relaxation ability. The relaxation ability was characterized by using the aging index of AI(E_IIM-STA_), AI(E_IIM-LTA_), AI(K_MRR-STA_), and AI(K_MRR-LTA_). Similarly, a linear regression was made between the rheological aging index and the one determined from initial instantaneous modulus and the modulus relaxation rate. As indicated by the correlation coefficient, R^2^, in Table 14, there was not a good relationship between the rheological aging index and the relaxation-related aging index. The highest correlation coefficient was found to be 0.3525 for the relationship between A_LTA_ and AI(K_MRR-LTA_). It indicated that the rheological changes of bitumen after aging were not necessarily consistent with relaxation ability.

Table 15 shows the relationship between the rheological aging index and the thermal cracking resistance. The thermal cracking resistance was characterized by the aging indexes including AI(E_SM-STA_), AI(E_SM-LTA_), AI(ε_UTS-STA_), and AI(ε_UTS-LTA_). Similarly, a linear regression between the rheological aging index and the one determined from stiffness modulus and the ultimate tensile strain. As indicated by the correlation coefficient, R^2^, in Table 15, a good relationship between the rheological aging index and the cracking related aging index were observed at −15 °C. However, similar relationships were not found at −25 °C. It may be due to the relatively small fraction of the hardening effect of aging at −15 °C when compared with the high stiffness modulus at extremely low temperatures. When the stiffness modulus was beyond 300 MPa at −25 °C, the bitumen binders were likely to be brittle and fracture under the direct tensile test. This may result in a weak relationship between the rheological aging index and low-temperature performance. 

## 4. Conclusions

In this study, the storage stability and aging resistance of slightly SBS polymer-modified bitumen binders were investigated by means of an FTIR spectroscopy test, a storage stability test, a DSR rheological test, a stress relaxation test, and a direct tensile test. The three types of slightly SBS polymer-modified bitumen binders have been applied for the asphalt concrete impermeable layer of PSPSs in China. Therefore, the results of this study would give better insight into the durability of slightly SBS polymer modified bitumen binders and establish a benchmark for the evaluation of durability-related issues of the actual asphalt concrete impermeable layer that applied in PSPSs. Based on the obtained results and the related analysis, the following conclusions were drawn:

The segregation indexes based on complex modulus and phase angle were proposed, and it was found that the change in the complex modulus was more sensitive to polymer segregation compared with phase angle. Different types of slightly SBS modified bitumen binders all had good storage stability, and even at a disadvantagous long-term period of 7 days of storage, the complex modulus and phase angle can remain fairly constant.

The slightly SBS-modified bitumen binders could be well identified by the plateau of phase angle master curves when compared with traditional virgin bitumen and normal polymer-modified bitumen. The change of the phase angle plateau after aging indicated the degradation of the SBS polymer as well as damage to the polymer network structure.

An FTIR analysis demonstrated that slightly SBS-modified bitumen exhibited serious aging of base bitumen together with higher polymer degradation. The aging indexes obtained from the carbonyl index and the polybutadiene (PB) index can well rank the aging resistance. 

The horizontal shift factor and the deviation area determined from the deviation degree of the complex modulus master curve were proposed as the rheological aging indexes. Both of these two indexes can generally give a consistent ranking for the aging resistance for various bitumen binders. Three types of slightly SBS modified bitumen binders exhibited inconsistent aging resistance when compared with virgin Karamay SG70 bitumen and normal polymer modified bitumen. 

The aging indexes based on the change of the initial instantaneous modulus and the modulus relaxation rate were proposed to explain the relaxation properties. Both of the aging indexes were sensitive to the type of bitumen and aging, as well as the test temperature. The reduction of the relaxation properties can be explained by the increase of the modulus together with the reduction of the modulus relaxation rate. After long-term aging, slightly SBS-modified bitumen binders seemed to lose more stress relaxation ability.

With respect to the direct tensile test, the aging indexes determined by the increase in the stiffness modulus and the loss of the ultimate tensile strain can be used to evaluate the susceptibility of bitumen aging. The minimum test temperature that can meet a tensile strain larger than 1%, together with a stiffness lower than 300 MPa, can be regarded as the brittle cracking temperature. Before and after aging, slightly SBS-modified bitumen binders generally exhibited a similar crack resistance to traditional virgin Karamay SG70 bitumen. 

An attempt was made to establish the relationship of the aging indexes between the FTIR analysis, rheological properties, and low-temperature performance. It was found that the relationship among these different aging indexes was weak. More fundamental studies are needed to bridge these gaps.

In general, slightly SBS-modified bitumen should be well-designed to obtain an obvious improvement on aging resistance as well as low-temperature performance. Highly modified bitumen is foreseen to be promising in the case of extremely low temperatures and long-term durability.

The purpose of this study was to obtain better insight into the storage stability and aging resistance of slightly SBS polymer-modified bitumen binders. Since the aging resistance and durability of bitumen binders can strongly affect the durability of asphalt concrete, the relationship of performance between the aged bitumen and the aged asphalt concrete should be investigated. In the future, more laboratory and field studies will need to be performed on the long-term durability of the asphalt concrete impermeable layer of PSPSs.

## Figures and Tables

**Table 1:** Summary of the unaged, short-term aged, and long-term aged bitumen binders.

Unaged Bitumen	Short-Term Aged Bitumen	Long-Term Aged Bitumen
Karamay SG70	Karamay SG70-STA	Karamay SG70-LTA
Jingbo SG70	Jingbo SG70-STA	Jingbo SG70-LTA
Jurong SG90	Jurong SG90-STA	Jurong SG90-LTA
Liaohe SG90	Liaohe SG90-STA	Liaohe SG90-LTA
Jingbo PMB	Jingbo PMB-STA	Jingbo PMB-LTA

**Table 2:** Aging indexes based on FTIR analysis of five bitumen binders.

Aging Index (%)	Type of Bitumen				
	Karamay SG70	Jingbo SG70	Jurong SG90	Liaohe SG90	Jingbo PMB
AI_C=O-STA_	18.9	43.2	56.3	72.7	15.4
AI_C=O-LTA_	59.3	840.5	213.1	194.1	81.1
AI_S=O-STA_	5.1	8.3	0.2	1.5	1.6
AI_S=O-LTA_	37.1	20.5	7.1	4.7	6.6
AI_PB-STA_	/	−10.0	−9.3	−10.8	−3.6
AI_PB-LTA_	/	−15.7	−19.7	−19.4	−6.2
AI_PS-STA_	/	−9.3	−9.7	−10.6	−0.7
AI_PS-LTA_	/	−16.0	−13.6	−14.5	−1.4

**Table 3:** Storage stability test results based on the difference in the softening point.

Softening Point (°C)	Type of Bitumen			
	Jingbo SG70	Jurong SG90	Liaohe SG90	Jingbo PMB
Unaged sample	52.2	50.7	59.5	62.6
Top section	53.0	50.9	61.2	63.9
Bottom section	52.5	50.8	60.8	63.2
Softening point difference (°C)	0.5	0.1	0.4	0.7

**Table 4:** Complex modulus segregation indexes of various bitumen binders at different storage times.

Segregation Index	Storage Time (d)	Temperature (°C)						
		34	40	46	52	58	64	70
SI_G*@Jingbo SG70_ (%)	2	0.128	0.169	0.240	0.368	0.649	0.765	1.071
	4	0.275	0.522	1.040	1.276	1.311	1.844	2.403
	7	1.675	1.747	2.098	2.178	2.303	3.069	3.238
SI_G*@Jurong SG90_ (%)	2	5.260	5.960	6.230	7.440	7.960	8.460	8.550
	4	8.709	8.990	10.260	10.950	11.120	11.230	11.560
	7	13.260	14.009	14.405	14.560	14.620	14.750	14.960
SI_G*@Liaohe SG90_ (%)	2	0.541	4.829	6.550	7.226	7.615	7.350	7.850
	4	4.432	6.560	7.860	7.981	8.020	8.500	8.750
	7	7.968	8.690	8.960	9.210	9.360	9.990	10.230
SI_G*@Jingbo PMB_ (%)	2	0.912	1.217	1.403	1.501	1.605	2.199	2.384
	4	1.500	1.700	2.500	2.900	3.400	6.135	7.000
	7	2.078	2.754	3.700	3.778	4.414	6.721	7.527

**Table 5:** Phase-angle segregation indexes of various bitumen binders at different storage times.

Segregation Index	Storage Time (d)	Temperature (°C)						
		34	40	46	52	58	64	70
SI_δ@Jingbo SG70_ (%)	2	0.167	0.150	0.148	0.122	0.090	0.088	0.079
	4	0.294	0.287	0.250	0.213	0.212	0.206	0.201
	7	0.553	0.517	0.477	0.450	0.417	0.384	0.309
SI_δ@Jurong SG90_ (%)	2	0.290	0.284	0.252	0.101	0.091	0.038	0.026
	4	0.680	0.645	0.638	0.603	0.566	0.535	0.456
	7	2.008	1.620	1.284	1.154	1.036	0.858	0.815
SI_δ@Liaohe SG90_ (%)	2	0.166	0.146	0.144	0.143	0.142	0.013	0.001
	4	0.381	0.359	0.325	0.303	0.266	0.221	0.199
	7	1.135	0.905	0.612	0.557	0.440	0.395	0.385
SI_δ@Jingbo PMB_ (%)	2	0.379	0.276	0.195	0.141	0.108	0.091	0.031
	4	0.865	0.807	0.795	0.705	0.658	0.534	0.454
	7	1.320	1.191	1.167	1.003	0.974	0.909	0.904

**Table 6:** The horizontal shift factor and deviation area of various bitumen binders.

Type of Bitumen	Aging Index					
	$\alpha_{STA}$	$\alpha_{LTA}$	$\alpha_{LTA}-\alpha_{STA}$	A_STA_	A_LTA_	A_LTA_ − A_STA_
Karamay SG70	0.23	1.50	1.27	0.81	3.48	2.67
Jingbo SG70	0.40	1.80	1.40	2.18	5.92	3.74
Jurong SG90	1.97	3.37	1.40	7.68	11.42	3.74
Liaohe SG90	0.12	1.20	1.08	0.60	3.89	3.29
Jingbo PMB	0.10	1.01	0.91	0.70	4.00	3.30

**Table 8:** Fitting results for the relaxation test at −25 °C.

Type of Bitumen	E_0_ [MPa]	E@_0.1s_ [MPa]	E@_1s_ [MPa]	E@_10s_ [MPa]	E@_600s_ [MPa]	K	R^2^
Karamay SG70	376.78	304.30	80.62	40.49	5.85	0.464	0.978
Karamay SG70-STA	384.55	306.42	143.09	60.63	7.03	0.432	0.981
Karamay SG70-LTA	389.87	311.07	158.12	60.89	7.40	0.398	0.990
Jingbo SG70	340.22	269.55	60.93	28.14	2.77	0.493	0.986
Jingbo SG70-STA	346.31	288.48	72.20	54.21	6.12	0.437	0.980
Jingbo SG70-LTA	554.35	291.6	133.90	40.42	7.67	0.362	0.983
Jurong SG90	496.02	304.9	81.48	39.47	4.48	0.484	0.977
Jurong SG90-STA	518.50	334.67	117.22	54.33	4.75	0.446	0.987
Jurong SG90-LTA	660.46	365.48	136.42	67.07	13.41	0.347	0.987
Liaohe SG90	399.00	20.09	7.19	2.78	0.23	0.541	0.975
Liaohe SG90-STA	420.31	32.11	10.12	3.56	1.23	0.432	0.982
Liaohe SG90-LTA	442.36	40.11	11.65	4.98	2.26	0.424	0.979
Jingbo PMB	321.84	176.92	56.87	17.46	1.57	0.548	0.996
Jingbo PMB-STA	334.20	204.53	76.95	26.15	2.64	0.503	0.992
Jingbo PMB-LTA	372.61	236.37	110.70	47.36	7.39	0.399	0.991

**Table 9:** The aging indexes based on the initial instantaneous modulus E_0_ and modulus relaxation rate K, using the unaged bitumen binder as the reference.

Test Temperature	Aging Index	Type of Bitumen				
		Karamay SG70	Jingbo SG70	Jurong SG90	Liaohe SG90	Jingbo PMB
−10 °C	AI(E_IIM-STA_)	1.098	1.032	1.137	1.084	1.101
	AI(E_IIM-LTA_)	1.113	1.219	1.282	1.390	1.385
	AI(K_MRR-STA_)	0.953	0.837	0.962	0.989	0.938
	AI(K_MRR-LTA_)	0.805	0.650	0.691	0.969	0.788
−25 °C	AI(E_IIM-STA_)	1.021	1.018	1.045	1.053	1.038
	AI(E_IIM-LTA_)	1.035	1.629	1.274	1.109	1.158
	AI(K_MRR-STA_)	0.931	0.886	0.921	0.799	0.918
	AI(K_MRR-LTA_)	0.858	0.734	0.717	0.784	0.728

**Table 10:** Direct tensile test results at −15 °C.

Type of Bitumen	Failure Mode	Ultimate Tensile Strength (MPa)	Ultimate Tensile Strain (%)	Stiffness Modulus (MPa)
Karamay SG70	Ductile	1.63	7.23	22.55
Karamay SG70-STA	Ductile	1.97	7.22	27.24
Karamay SG70-LTA	Brittle	2.71	2.00	135.30
Jingbo SG70	Ductile	2.36	7.23	33.40
Jingbo SG70-STA	Ductile	2.98	7.05	41.24
Jingbo SG70-LTA	Brittle	2.99	2.66	112.47
Jurong SG90	Ductile	2.81	8.41	33.38
Jurong SG90-STA	Brittle	2.58	2.36	109.52
Jurong SG90-LTA	Brittle	2.96	2.01	147.29
Liaohe SG90	Ductile	2.68	4.76	56.29
Liaohe SG90-STA	Ductile	2.61	3.70	70.40
Liaohe SG90-LTA	Brittle	2.73	1.71	159.76
Jingbo PMB	Ductile	0.80	7.40	10.91
Jingbo PMB-STA	Ductile	1.35	7.35	18.39
Jingbo PMB-LTA	Ductile	1.85	7.31	24.97

**Table 11:** Direct tensile test results at −25 °C.

Type of Bitumen	Failure Mode	Ultimate Tensile Strength (MPa)	Ultimate Tensile Strain (%)	Stiffness Modulus (MPa)
Karamay SG70	Brittle	2.75	0.90	304.57
Karamay SG70-STA	Brittle	2.82	0.85	330.80
Karamay SG70-LTA	Brittle	2.86	0.79	362.33
Jingbo SG70	Brittle	2.21	0.65	338.26
Jingbo SG70-STA	Brittle	2.18	0.62	352.03
Jingbo SG70-LTA	Brittle	2.15	0.59	366.84
Jurong SG90	Brittle	2.83	0.76	371.66
Jurong SG90-STA	Brittle	2.87	0.70	407.95
Jurong SG90-LTA	Brittle	3.17	0.68	463.23
Liaohe SG90	Brittle	3.20	1.31	243.36
Liaohe SG90-STA	Brittle	3.35	1.19	280.90
Liaohe SG90-LTA	Brittle	4.04	1.11	364.58
Jingbo PMB	Brittle	2.52	1.12	224.87
Jingbo PMB-STA	Brittle	2.73	1.02	268.88
Jingbo PMB-LTA	Brittle	3.09	0.88	350.51

**Table 12:** The aging indexes determined by the relative change in the stiffness modulus and ultimate tensile strain.

Test Temperature	Aging Index	Type of Bitumen				
		Karamay SG70	Jingbo SG70	Jurong SG90	Liaohe SG90	Jingbo PMB
−15 °C	AI(E_SM-STA_)	1.208	1.235	3.281	1.251	1.686
	AI(E_SM-LTA_)	6.000	3.367	4.413	2.838	2.289
	AI(ε_UTS-STA_)	0.999	0.975	0.281	0.777	0.993
	AI(ε_UTS-LTA_)	0.277	0.368	0.239	0.204	0.988
−25 °C	AI(E_SM-STA_)	1.086	1.041	1.098	1.154	1.196
	AI(E_SM-LTA_)	1.190	1.084	1.246	1.498	1.559
	AI(ε_UTS -STA_)	0.944	0.954	0.921	0.908	0.911
	AI(ε_UTS-LTA_)	0.878	0.908	0.895	0.847	0.786

**Table 13:** Relationship between the FTIR aging index and the rheological aging index.

Aging Index	AI_C=O-STA_	AI_C=O-LTA_	AI_PB-STA_	AI_PB-LTA_
$\alpha_{STA}$	y = 10.419x + 35.424 R^2^ = 0.1149	/	y = −0.9028x − 7.8404 R^2^ = 0.0605	/
$\alpha_{LTA}$	/	y = 47.952x + 192.46 R^2^ = 0.0196	/	y = −3.3846x − 9.0055 R^2^ = 0.3309
A_STA_	y = 2.7855x + 34.631 R^2^ = 0.1189	/	y = −0.2471x − 6.8665 R^2^ = 0.0711	/
A_LTA_	/	y = 16.526x + 182.73 R^2^ = 0.0289	/	y = −10.675x + 22.3 R^2^ = 0.1895

**Table 14:** Relationship between the rheological aging index and the relaxation ability.

Test Temperature	Aging Index	AI(E_IIM-STA_)	AI(E_IIM-LTA_)	AI(K_MRR-STA_)	AI(K_MRR-LTA_)
−10 °C	$\alpha_{STA}$	y = 0.0279x + 1.0747 R^2^ = 0.3392	/	y = 0.009x + 0.9307 R^2^ = 0.015	/
	$\alpha_{LTA}$	/	y = −0.0271x + 1.326 R^2^ = 0.0476	/	y = −0.0755x + 0.9146 R^2^ = 0.329
	A_STA_	y = 0.0066x + 1.0747 R^2^ = 0.2725	/	y = 0.0009x + 0.9336 R^2^ = 0.0022	/
	A_LTA_	/	y = −0.0002x + 1.2789 R^2^ = 3 × 10^−5^	/	y = −0.0216x + 0.9049 R^2^ = 0.3354
−25 °C	$\alpha_{STA}$	y = 0.0048x + 1.0323 R^2^ = 0.0635	/	y = 0.0229x + 0.8781 R^2^ = 0.1134	/
	$\alpha_{LTA}$	/	y = 0.0731x + 1.1112 R^2^ = 0.0865	/	y = −0.0242x + 0.8072 R^2^ = 0.1516
	A_STA_	y = 0.0011x + 1.0323 R^2^ = 0.0508	/	y = 0.0059x + 0.8769 R^2^ = 0.1084	/
	A_LTA_	/	y = 0.0253x + 1.0955 R^2^ = 0.129	/	y = −0.0105x + 0.8243 R^2^ = 0.3525

**Table 15:** Relationship between the rheological aging index and the thermal cracking resistance.

Test Temperature	Aging Index	AI(E_SM-STA_)	AI(E_SM-LTA_)	AI(ε_UTS-STA_)	AI(ε_UTS-LTA_)
−15 °C	$\alpha_{STA}$	y = 1.056x + 1.1366 R^2^ = 0.8936	/	y = −0.3577x + 1.0068 R^2^ = 0.8574	/
	$\alpha_{LTA}$	/	y = 0.5857x + 2.7412 R^2^ = 0.1409	/	y = −0.1586x + 0.6969 R^2^ = 0.2094
	A_STA_	y = 0.2751x + 1.0736 R^2^ = 0.8779	/	y = −0.0924x + 1.0263 R^2^ = 0.8287	/
	A_LTA_	/	y = 0.0648x + 3.4094 R^2^ = 0.0214	/	y = −0.0297x + 0.5858 R^2^ = 0.0911
−25 °C	$\alpha_{STA}$	y = −0.0225x + 1.1277 R^2^ = 0.0874	/	y = −0.0011x + 0.9282 R^2^ = 0.0017	/
	$\alpha_{LTA}$	/	y = −0.1056x + 1.5029 R^2^ = 0.2364	/	y = 0.0328x + 0.8045 R^2^ = 0.4033
	A_STA_	y = −0.0064x + 1.1304 R^2^ = 0.103	/	y = −9E−05x + 0.9278 R^2^ = 0.0002	/
	A_LTA_	/	y = −0.0208x + 1.4351 R^2^ = 0.1143	/	y = 0.0073x + 0.821 R^2^ = 0.2462

## Data Availability

Not applicable.
